# Spatial and viewpoint selectivity for others’ observed actions in monkey ventral premotor mirror neurons

**DOI:** 10.1038/s41598-017-08956-1

**Published:** 2017-08-15

**Authors:** Monica Maranesi, Alessandro Livi, Luca Bonini

**Affiliations:** 1 Istituto Italiano di Tecnologia (IIT), Brain Center for Social and Motor Cognition (BCSMC), Via Volturno 39, 43125 Parma, Italy; 2 Dipartimento di Neuroscienze, Università di Parma, Via Volturno 39, 43125 Parma, Italy

## Abstract

The spatial location and viewpoint of observed actions are closely linked in natural social settings. For example, actions observed from a subjective viewpoint necessarily occur within the observer’s peripersonal space. Neurophysiological studies have shown that mirror neurons (MNs) of the monkey ventral premotor area F5 can code the spatial location of live observed actions. Furthermore, F5 MN discharge can also be modulated by the viewpoint from which filmed actions are seen. Nonetheless, whether and to what extent MNs can integrate viewpoint and spatial location of live observed actions remains unknown. We addressed this issue by comparing the activity of 148 F5 MNs while macaque monkeys observed an experimenter grasping in three different combinations of viewpoint and spatial location, namely, lateral view in the (1) extrapersonal and (2) peripersonal space and (3) subjective view in the peripersonal space. We found that the majority of MNs were space-selective (60.8%): those selective for the peripersonal space exhibited a preference for the subjective viewpoint both at the single-neuron and population level, whereas space-unselective neurons were view invariant. These findings reveal the existence of a previously neglected link between spatial and viewpoint selectivity in MN activity during live-action observation.

## Introduction

The most widely known and distinctive feature of mirror neurons (MNs) is their capacity to become active when the subject performs an action as well as when he/she observes a similar action performed by another agent^1^.

Converging experimental evidence indicates that MNs can play a crucial role in action recognition^2^, and recent findings have shown that their discharge can be strongly modulated by both contextual and perceptual factors^3^. For example, MN discharge can be influenced by the viewpoint from which an action is observed^4^ as well as by its distance from the observer^5, 6^ or the contextual situation in which it occurs^7–9^. In everyday life, all these factors are usually combined in different ways: for example, if we are observing another’s action from a subjective viewpoint, such as when someone shows us how to play a piano chord, this action *necessarily* occurs within our peripersonal space. On the other hand, an action observed from an allocentric viewpoint can occur either within or outside our peripersonal space, with different implications for the possibility of interacting with the observed agent. Despite the importance of understanding how these factors can interact, previous neurophysiological studies have focused only on single factors in isolation. Indeed, MN viewpoint selectivity has been demonstrated with filmed actions presented on a screen^4^, that is, in a somehow ‘virtual’ space, whereas the spatial selectivity of MNs has been investigated with live actions presented from a single point of view, namely, frontal^5^ or lateral^6, 10^.

**Figure 1:**
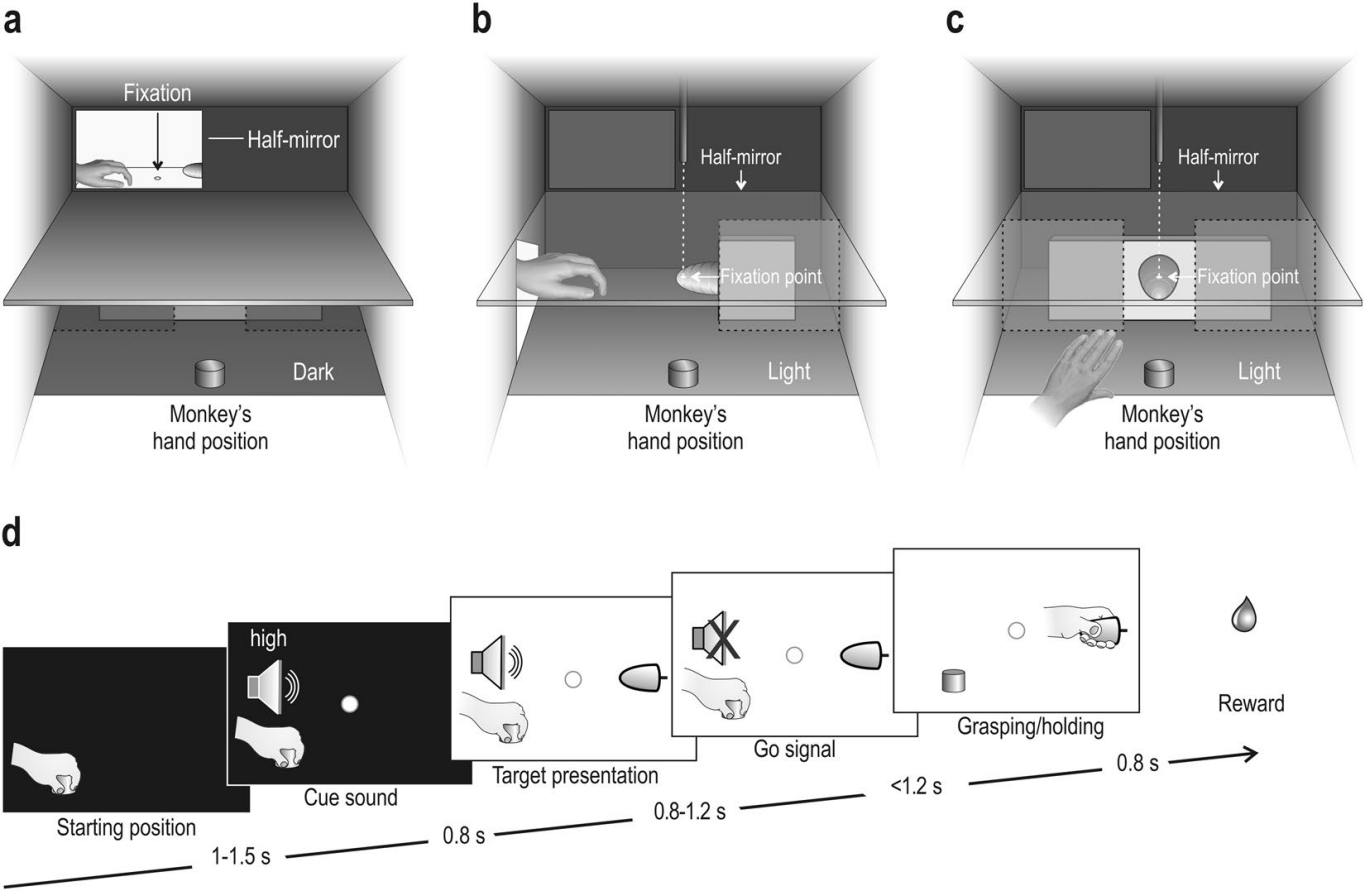
Apparatus, experimental conditions, and phases of the task. **(a–c)** Box and apparatus for presenting the monkey with experimenter’s action in three different combinations of viewpoint and spatial location: lateral view in the **(a)** extrapersonal space, and **(b)** peripersonal space, and **(c)** subjective view in the peripersonal space. **(d)** Task phases are the same for all conditions.

As a consequence, to date no study has directly investigated whether and to what extent viewpoint and spatial location of an observed action are integrated in the visual response of ventral premotor (area F5) MNs. To address this issue, we recorded F5 MNs while monkeys performed grasping actions and when they observed a similar action performed by an experimenter with three different combinations of viewpoint and spatial location (see Fig. 1): (1) lateral view in the extrapersonal space, (2) lateral view in the peripersonal space, and (3) subjective view in the peripersonal space.

## Results

We recorded 155 mirror neurons (MNs) from area F5. A few of them (7/155, 4.5%) showed suppressed activity during action observation and, due to their low number, have not been included in the subsequent analyses. This paper therefore focuses on a set of 148 MNs.

**Figure 2:**
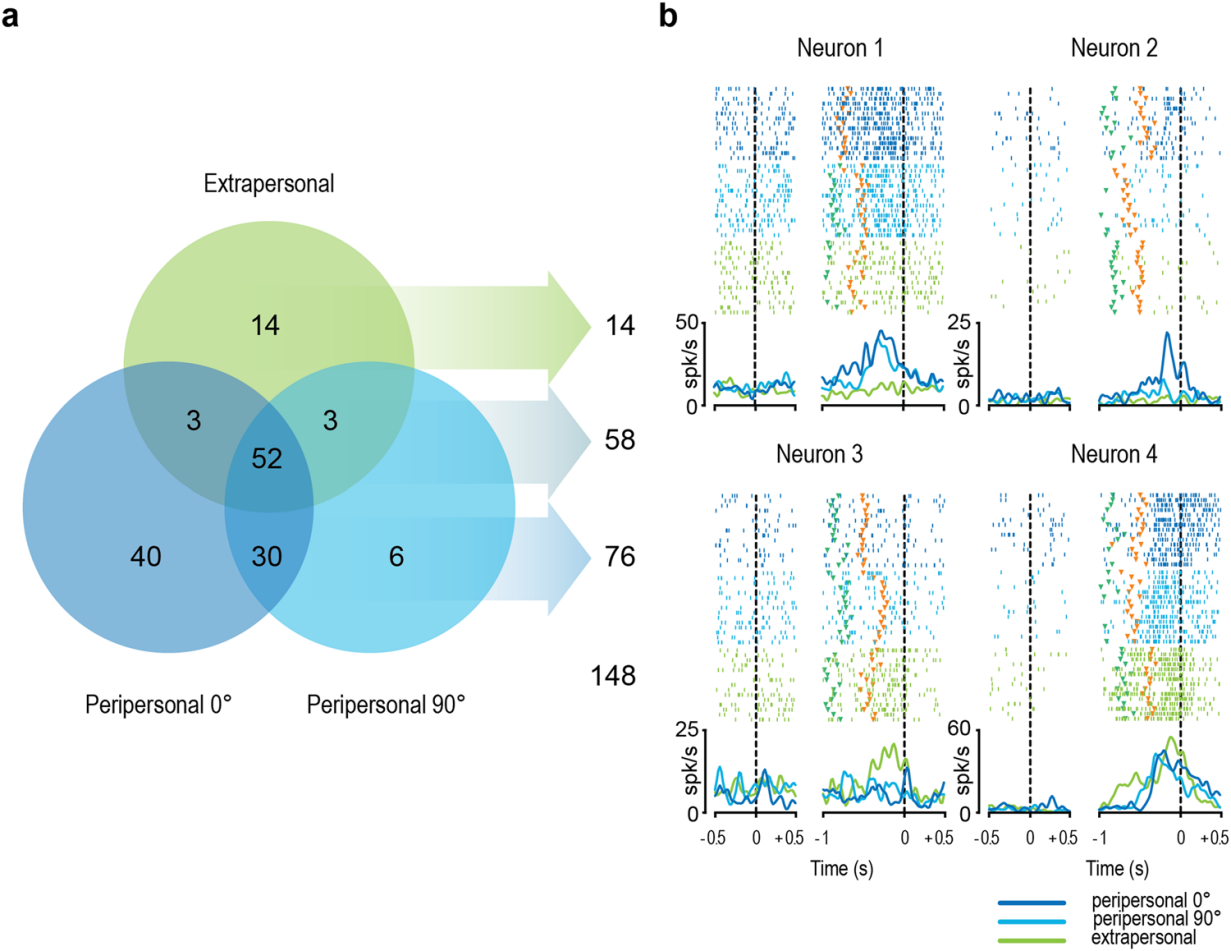
Spatial and/or viewpoint selectivity of the recorded MNs. **(a)** Venn diagram illustrating the number of MNs significantly activated during single or multiple conditions. **(b)** Examples of MNs with different spatial and viewpoint selectivity. For each neuron, rasters and spike-density function are aligned (dashed lines) on object presentation (left part) and, after the gap, on the beginning of object-pulling phase (right part). Markers: green, end of the cue sound (go-signal); orange, detachment of the experimenter’s hand from the starting position.

Figure 2a illustrates the distribution of the recorded neurons in terms of spatial and/or viewpoint selectivity. The majority of them (76/148, 51.4%) discharged stronger when the observed action occurred in the peripersonal space. Among them, a greater number showed selectivity for the subjective (40/76, 52.6%) relative to lateral (6/76, 7.9%) viewpoint, whereas the remaining were view-invariant (30/76, 39.5%). Only 14 out of 148 MNs (9.4%) showed a preference for the observation of actions in the extrapersonal space, whereas all the remaining MNs (58/148, 39.2%) were space-unselective and, in most cases (52/58, 89.7%), were view-invariant as well. It is interesting to note that even disregarding MNs discharging only for the subjective viewpoint (which cannot be tested in the extrapersonal space), a greater number of the remaining neurons showed a preference for the peripersonal (36/108, 33.3%) relative to the extrapersonal (17/108, 15.7%) space. On the other hand, disregarding MNs discharging only in the extrapersonal space (for which the subjective viewpoint cannot be tested), a larger number of MNs showed a preference for the subjective (43/134, 32.1%) relative to the lateral (9/134, 6.7%) viewpoint.

Figure 2b provides examples of the most representative types of MNs. Neuron 1 became active only when the observed action occurred in the peripersonal space, and it showed view-invariant activation. Neuron 2 is a peripersonal MN as well, but it also showed selective activation when the monkey observed the action from a subjective viewpoint. Neuron 3 discharged only during observation of the action carried out in the extrapersonal space, whereas Neuron 4 exemplifies a view-and-space-invariant response.

**Figure 3:**
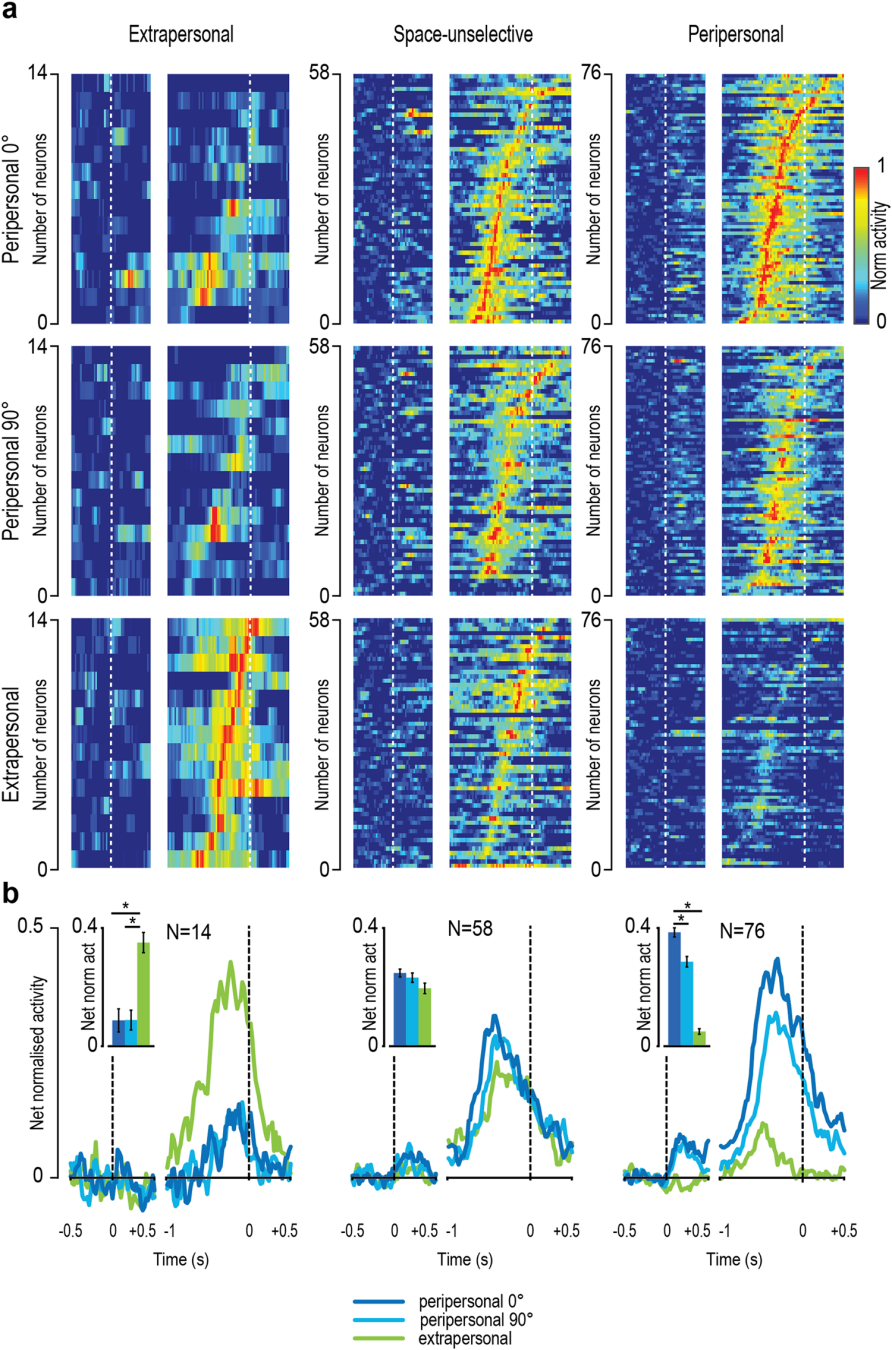
Population dynamic of MNs with different spatial selectivity during action observation in the three experimental conditions. (**a**) Normalised raw activity (no baseline subtraction) of each set of MNs. Each row represents a single neuron. In each panel neurons have been ordered based on the timing of their peak of activity (earliest on bottom) within that condition. White dashed lines represent the different alignment events: first (on the left, before the gap) object presentation, second (on the right, after the gap) object-pulling onset. (**b**) Net normalised population activity of each set of MNs. Black dashed lines correspond to the same alignment events described in (**a**). Histograms show the average (±1 standard error) of each population activity for each condition. *P < 0.05.

To better scrutinise the relationship between viewpoint and spatial selectivity of MNs response, we subdivided all the recorded neurons into three populations: extrapersonal, space-unselective, and peripersonal MNs - depending on their spatial tuning (see rightward column in Fig. 2a). We then investigated the possible modulation of each population activity depending on the viewpoint (Fig. 3). Extrapersonal MNs showed a stronger response during action observation in the extrapersonal space relative to both viewpoints tested in the peripersonal space, which in turn did not differ from each other. Similarly, viewpoint selectivity of space-unselective neurons did not reach statistical significance (P < 0.01) neither at the population level (F(2, 114)_ = _3.7, P = 0.03) nor in terms of single unit peak of activity (in spike/second) (subjective: 47 ± 3.9; lateral: 45.6 ± 5.3; t = 0.52, P = 0.64). In contrast, peripersonal MNs showed stronger tuning to subjective relative to lateral viewpoint both in terms of intensity of population activity (F(2, 150) = 183.1, P < 0.001) and of single unit peak of activity (subjective: 42.5 ± 3.1; lateral: 38.4 ± 3.1; t = 3.35, P = 0.0013).

## Discussion

In this study we investigated the interaction between spatial and viewpoint selectivity of MN responses during the observation of live actions. The findings revealed a stronger tuning of MN activity for the peripersonal relative to the extrapersonal space and, within the peripersonal space, a preference for actions observed from a subjective viewpoint, suggesting the existence of a functional link between the processing of spatial location and viewpoint of observed actions.

The majority of MNs (60.8%) showed space-selective visual response, with a strong preference (84.4%) for the peripersonal space. In a previous study^5^, by contrast, Caggiano and coworkers found that half of the recorded MNs were space-unselective, whereas the other half were evenly distributed between peripersonal- and extrapersonal-space selectivity. A plausible explanation for this discrepancy is that, in the previous study, peripersonal and extrapersonal spatial sectors were relatively close and physically contiguous (28 and 48.5 cm, respectively, with a border of the reaching space at about 37 cm). In contrast, in our experimental setting, extrapersonal actions occurred at 60 cm from the monkey and behind a transparent barrier, which is known to emphasize the dichotomy between peripersonal and extrapersonal space^5^. As previously shown for peripersonal-space-constrained representation of objects in the motor cortex^6, 11^, the present findings extend the relevance of the peripersonal space as a source of information on others’ observed actions, supporting the idea that an important function of MNs may consist in preparing behavioural reactions during social interactions^5, 10^.

As compared to spatial coding, viewpoint selectivity characterised a smaller set of MNs (42.6%). This latter percentage is lower relative to the one reported by the study of Caggiano and coworkers^4^ with filmed actions (74.1%), likely because we tested two rather than three different viewpoints. Unfortunately, our setup did not allow us to test neuronal responses from a frontal viewpoint, which would have made more straightforward the comparison of our results with previous data. Nonetheless, it is interesting to note that we found a significant preference for the processing of actions seen from a subjective viewpoint in terms of number of single neurons, magnitude of population activity, and single-unit peak of activity only in the subpopulation of peripersonal MNs. It might be argue that the subjective view preference may simply be accounted for by the higher proximity of the experimenter to the monkey, which may induce a higher level of arousal. However, this latter would likely result in a greater EMG activation, which we can exclude during observation of actions from both subjective^10^ and lateral^7^ viewpoints. Moreover, in the absence of EMG activation, any possible difference in the level of arousal among conditions should influence neuronal activity in an unspecific way, that is, equally affecting baseline, object presentation and action observation epochs: this is not the case as clearly shown by single neuron examples (see Fig. 2) and population activity (see Fig. 3), thus indicating that the prevalence of subjective viewpoint selectivity cannot be accounted for by unspecific factors. A further support to this argument is that a prevalence of subjective-view preference relative to the other tested views was also found by Caggiano and coworkers with filmed actions. Although this effect did not reach significance at the single-unit level^4^, it clearly emerged when LFP were considered^12^, suggesting that a subjective view of another’s action can provide a stronger synaptic input to premotor MNs. Therefore, based on our data, it can be proposed that this input is turned into a relevant increase in the firing rate of MNs only when they are specifically tuned to information coming from the observer’s peripersonal space.

Human data support the relevance of the functional link between the viewpoint and spatial location of observed actions. Indeed, when actions are displayed on a screen, they either evoke similar motor activation regardless of the viewpoint from which they are observed^13^ or, most often, induce an increased motor resonance when seen from a subjective perspective^14–17^. Furthermore, human studies using a variety of techniques have provided converging evidence that motor resonance is stronger in ‘the here and now’, that is, during live presentation of actions compared to presentation of videos with the same motor content^18–20^, as previously demonstrated in monkeys^4^. The single-neuron evidence of the present study contributes to this picture by showing that MN preference for peripersonal space and subjective view of live actions are closely related with each other.

## Conclusions

In real-life situations, observing a hand action from a subjective viewpoint invariably implies that it is embedded into the observer’s peripersonal space, regardless of whether the acting hand belongs to the observer or to another individual. Previous studies have shown that own-hand visual feedback retains a particular relevance for MN activity in both parietal^21, 22^ and premotor^10^ areas, suggesting that visual information on acting hands coming from the peripersonal space may have at least a dual function, depending on both the viewpoint and contextual situation: indeed, it may serve as a prompt in joint actions and social interactions^5, 23^ or as feedback with which to monitor own action, such as during motor learning processes^24, 25^. The demonstration that MN discharge can be modulated by different factors, such as spatial location and viewpoint, should prompt researchers to undertake future studies of the possible functions of similar forms of multidimensional tuning^2^ in different, and possibly more natural, contextual situations.

## Methods

### Subjects

Experiments were carried out on two male adult monkeys: M1 (*Macaca nemestrina*, 9 kg) and M2 (*Macaca mulatta*, 7 kg). Both animals were trained to perform the task described below with the hand (left) contralateral to the hemisphere to be recorded (right). After training was completed, a head-fixation system and a recording chamber were implanted under general anaesthesia (ketamine hydrochloride, 5 mg/Kg i.m., and medetomidine hydrochloride, 0.1 mg/Kg i.m.), followed by postsurgical pain medications. Surgical procedures were the same as previously described^26^. All experimental protocols complied with the European law on the humane care and use of laboratory animals (directives 86/609/EEC, 2003/65/CE, and 2010/63/EU), they were authorised by the Italian Ministry of Health (D.M. 294/2012-C and 11/12/2012), and were approved by the Veterinarian Animal Care and Use Committee of the University of Parma (Prot. 78/12 17/07/2012).

#### Apparatus and behavioural paradigm

Monkeys were trained to perform a go/no-go visuomotor task (execution task) in order to verify the presence of grasping-related activity (see^10^ for details on the behavioural paradigm), but in this paper we only focused on the go trials. Furthermore, the monkey observed the experimenter performing, with the left hand, the same task (observation task) constituted by three different experimental conditions (run in block): (1) observation of action in the extrapersonal space (about 60 cm from the monkey) from a lateral point of view (Figs 1a and 2) observation of action in the peripersonal space (about 25 cm from the monkey) from a lateral point of view (Figs 1a and 3) observation of action in the peripersonal space from a subjective point of view. During observation of actions in the peripersonal space the target was placed in the same absolute position regardless of the viewpoint, at a reaching distance from the monkey’s hand. In particular, for the subjective viewpoint condition, the experimenter, standing behind and on the left of the monkey, performed the task starting from an initial position located 10 cm to the left of the monkey’s left hand. In turn, the monkey was required to remain still with its hand on the starting position during the entire duration of each action observation trial to get the reward. Some of these data have been employed in previous studies^6, 10^.

A schematic drawing of the apparatus used for the task is shown in Fig. 1. All the conditions of the task (both in the execution and observation mode) were characterised by the same temporal sequence of events (Fig. 1d), as follows. A fixation point was presented in the exact position of the (not yet visible) target object in complete darkness, and the monkey was required to start fixating on it within 1.2 s. Fixation onset determined the presentation of a cue sound (a 1200 Hz sine wave). After 0.8 s, the target object became visible, and after a variable time lag (0.8–1.2 s), the sound ceased (go signal). At this point, the experimenter reached, grasped, and pulled (for at least 0.8 s) the object, while the monkey had to remain still and to maintain fixation until the reward was delivered.

LabView-based software monitored the task’s stages: if the monkey broke fixation, made an incorrect movement, or did not respect the task’s temporal constraints, the trial was aborted and no reward was delivered. After the monkey correctly performed a trial, a fixed amount of juice was delivered automatically (pressure reward delivery system, Crist Instruments).

### Recording techniques

Neuronal recordings were performed by means of multielectrode linear arrays (16-channel U-probes, Plexon, and 16-channel silicon probes^27, 28^, distributed by ATLAS Neuroengineering, Belgium), with impedance (measured at 1 kHz) ranging from 0.3 to 1.5 MΩ. Devices and techniques employed for probe insertions have been described elsewhere^6, 29^. The signal was amplified and sampled at 40 KHz with a 16-channel Omniplex system (Plexon), and all final quantitative analyses were performed offline, as described below.

#### Recording of behavioural events and definition of epochs of interest

Contact sensitive devices (Crist Instruments) were used to detect when the monkey or the experimenter touched with the hand the metal surface of the starting position or the target object. A switch located behind the object signaled the onset of the object-pulling phase. TTL signals were used by LabView-based software to monitor the monkey’s and the experimenter’s performance, to control the presentation of auditory and visual cues of the behavioural paradigm, and to control the reward delivery. All these signals were fed to the recording system to be stored in parallel with neuronal activity for subsequent analyses.

Eye position was monitored with an eye-tracking system consisting of a 50 Hz CCD video camera (Ganz, F11CH4) equipped with an infrared filter and two spots of infrared light. An analog signal related to horizontal and vertical eye position was fed to a computer equipped with dedicated software (Pupil), enabling calibration and basic processing of eye-position signals. The monkey was required to maintain its gaze on the fixation point (tolerance radius 5°) during all the tasks.

Different epochs of interest were defined for single-neuron analyses, as follows: (1) baseline, 500 ms before object presentation, (2) object presentation, from 50 to 450 ms after switching on the light, (3) hand shaping, from the detachment of the hand from the starting position to the beginning of object pulling, and (4) object holding, from pulling onset to 500 ms after this event. The same epochs were used to analyse neuronal responses during both the execution task and all conditions of the observation task.

In previous studies with the same monkeys and tasks, we showed that there was no significant forelimb EMG activation during observation of action, both from a lateral view^7^ and from a subjective view^10^, in line with the existing literature^30, 31^.

### Analyses of neuronal data

#### Single-neuron analyses

The raw signals of all trials of a recording session was high-pass filtered (300 Hz), and single-unit action potentials were sorted offline (Plexon) using principal-component and template-matching techniques, as previously described^6^.

Preliminary analyses were carried out in order to identify neurons with mirror properties. First, we applied a one-way repeated measures ANOVA (four levels of the factor Epoch, P < 0.01, possibly followed by Newman-Keuls post hoc tests, P < 0.05) to each neuron response in the execution task and in each condition of the observation task, separately. All neurons showing stronger discharge during the hand-shaping and/or object-holding epoch relative to both baseline and object-presentation epochs in the execution task were classified as grasping neurons (note that, in this way, the grasping-related response cannot be accounted for by simple object-related activity). The same analysis was carried out for each condition of the observation task: all grasping neurons showing grasping-related activity also during at least one of the conditions of the observation task were classified as mirror neurons. All neurons with responses weaker than 5 spike/second across all the analysed epochs were not included in the final data set.

In order to classify MNs based on their space and viewpoint selectivity, the discharge of each of them has been further analysed with a 3 × 2 repeated measures ANOVA (factors: Condition and Epoch, P < 0.01). The three levels of the factor Condition corresponded to the three conditions of the observation task (extrapersonal space, peripersonal space – lateral viewpoint, peripersonal space – subjective viewpoint). The two levels of the factor Epoch corresponded to object presentation (in order to exclude that possible selectivity could be accounted for by the vision of the object rather than of the action) and the 500 ms preceding pulling onset. Neurons selectivity for the three tested conditions were assessed only if significant effects of both factors were found (either isolated or in interaction). By means of Newman-Keuls post-hoc tests (p < 0.05) we classified neurons as 1) extrapersonal, 2) peripersonal-lateral viewpoint, or 3) peripersonal-subjective viewpoint selective, when a single condition significantly differed from both the others. When the post-hoc test evidenced a similar response in two conditions relative to the third one, neurons were classified according to their mixed selectivity. When only a main effect of the factor Epoch was found, neurons were classified as unselective, whereas neurons showing only a significant main effect of the factor Condition were excluded from the data set.

#### Population analyses

Population analyses were performed that took into account single-neuron responses expressed in terms of net-normalised mean activity^32^. For each neuron, the mean activity was calculated in bins of 20 ms across trials of all conditions of the observation task. Then, the absolute highest activity value was used as the divisor of the value of each single bin in all conditions (normalised mean activity, ranging from 0 to 1). The average response of the neuron during the baseline period was finally subtracted from that of each bin, providing the net-normalised vector.

The population response was analysed (during the 500 ms epoch preceding pulling onset) with a one-way repeated measures ANOVAs (factors: condition, P < 0.01), followed by Newman-Keuls post-hoc tests (P < 0.05), and plotted by averaging activity across neurons in bins of 60 ms.
